# Two-Hop Energy Consumption Balanced Routing Algorithm for Solar Insecticidal Lamp Internet of Things

**DOI:** 10.3390/s22010154

**Published:** 2021-12-27

**Authors:** Xuanchen Guo, Lei Shu, Xing Yang, Edmond Nurellari, Kailiang Li, Bangsong Du, Heyang Yao

**Affiliations:** 1 College of Engineering, Nanjing Agricultural University, Nanjing 210031, China; 2019112039@njau.edu.cn (X.G.); 2019212014@njau.edu.cn (X.Y.); 2019812109@njau.edu.cn (B.D.); 2020212015@njau.edu.cn (H.Y.); 2 College of Artificial Intelligence, Nanjing Agricultural University, Nanjing 210031, China; kailiang_li@njau.edu.cn; 3 College of Engineering, University of Lincoln, Lincoln LN6 7TS, UK; ENurellari@lincoln.ac.uk

**Keywords:** energy consumption balanced routing, Solar Insecticidal Lamps Internet of Things, geographic forwarding, two-hop information

## Abstract

Due to the sparsity deployment of nodes, the full connection requirement, and the unpredictable electromagnetic interference on communication caused by high voltage pulse current of Solar Insecticidal Lamps Internet of Things (SIL-IoTs), a Two-Hop Energy Consumption Balanced routing algorithm (THECB) is proposed in this research work. THECB selects next-hop nodes according to 1-hop and 2-hop neighbors’ information. In addition, the greedy forwarding mechanism is expressed in the form of probability; that is, each neighbor node is given a weight between 0 and 1 according to the distance. THECB reduces the data forwarding traffic of nodes whose discharge numbers are relatively higher than those of other nodes so that the unpredictable electromagnetic interference on communication can be weakened. We compare the energy consumption, energy consumption balance, and data forwarding traffic over various discharge numbers, network densities, and transmission radius. The results indicate that THECB achieves better performance than Two-Phase Geographic Greedy Forwarding plus (TPGFPlus), which ignores the requirement of the node-disjoint path.

## 1. Introduction

**Figure 1:**
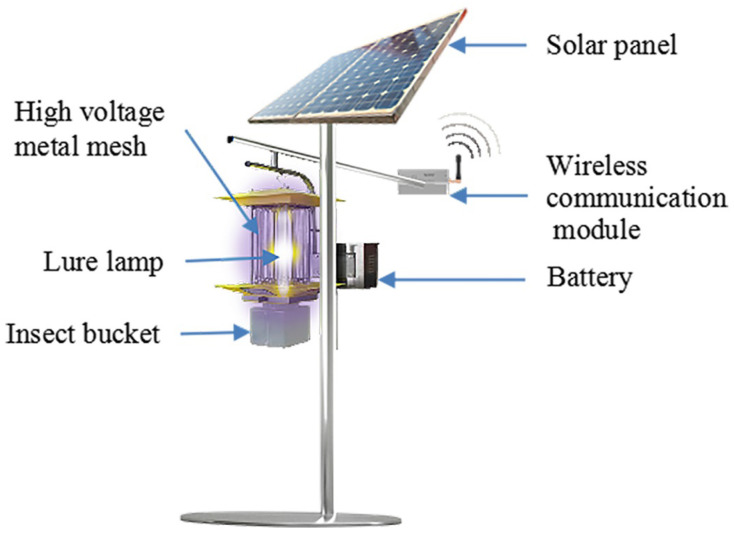
The SIL attracts migratory pests via a lure lamp in the evening and kills them by releasing high voltage pulse currents generated by the high voltage metal mesh. The dead pests are sorted in the insert bucket. The battery supplies the energy for the SIL. After being equipped with the wireless communication module, the SIL node can send its data to other nodes.

Recently, to achieve accurate information perception, agricultural Internet of Things (IoT) devices have been widely used in various smart agriculture scenarios [1]. For instance, Solar Insecticidal Lamps Internet of Things (SIL-IoTs) is an innovative agricultural IoTs that extends IoTs technology towards the Solar Insecticidal Lamp (SIL), which kills pests through generating high voltage discharges (2150 V to 6000 V) and contributes to (1) pest outbreak area location, (2) pesticide dosage reduction, and (3) environmental conditions monitoring [2]. The main hardware components of SIL are shown in Figure 1.

At present, various SIL-IoTs products mainly adopt mobile cellular network (GSM) for data transmission, which leads to huge data traffic costs [3]. Besides, the large-scale deployment of SIL-IoTs nodes requires high communication quality and signal coverage of the network. However, it is uncertain that the remote area where SIL-IoTs is deployed is covered by a 4G or 5G network. Hence, an alternative approach for transmitting data must be adopted for SIL-IoTs. Notably, the internode distance is smaller than the communication range of ZigBee; thus, it is feasible for the SIL-IoTs to communicate with each other via applying ZigBee technology [2]. Therefore, the routing problem (an algorithm that defines how exactly to route a packet from the source node to the sink) of the SIL-IoTs can be transformed into the routing problem of traditional Wireless Sensor Networks (WSNs). Geographic routing is widely used in WSNs, where packets are forwarded locally and greedily to 1-hop neighbor closest to the sink [4]. Each sensor node forwards data only through its neighbors’ information in the distributed routing approach.

The same as WSNs nodes, SIL-IoTs nodes are powered by a battery with limited capacity. Even though the SIL-IoTs node is equipped with the solar panel, due to (1) the dependence on long-term exposure to the sunlight to collect energy, the solar panel would supplement the battery with very little energy in continuous cloudy days or rainy areas, and (2) keeping the lure lamp on to attract pests and releasing high voltage pulse currents to kill pests both cost much energy, which makes the battery consumed faster in SIL-IoTs than that in traditional WSNs, it is still needed to design a novel routing algorithm for SIL-IoTs.Besides, it is worth noting that high voltage discharges of the SIL have a great impact on data transmission [5,6]. Moreover, different discharge numbers of different SIL-IoTs nodes lead to inconsistent residual energy of nodes.

To the best of our knowledge, no study takes communication energy management and the unpredictable electromagnetic interference on the communication of SIL-IoTs into consideration, which has the following features:

- The large-scale and sparse network.
- Full connection requirement and fixed locations of nodes.
- Unpredictable electromagnetic interference on communication caused by high voltage discharges.

Therefore, we present a novel Two-Hop Energy Consumption Balanced (THECB) routing algorithm to improve the energy consumption balance and reduce unpredictable electromagnetic interference on the communication of the SIL-IoTs. The main contributions of this article are shown in follows:

- Because high voltage discharges of the SIL impact communication, a Two-Hop Energy Consumption Balanced (THECB) routing algorithm is proposed to reduce data forwarding traffic of the nodes whose discharge numbers are relatively higher than those of other nodes. Therefore the unpredictable electromagnetic interference in communication can be weakened.
- Considering the energy consumption of the discharges in the SIL, reducing the data forwarding traffic of the nodes whose discharge numbers are relatively higher than those of other nodes can also improve the energy consumption balance since the energy consumed by communication of these nodes is reduced. Besides, although not using these nodes for data transmission may cause longer routing paths, the results indicate that THECB can still reduce the energy consumption and improve the energy consumption balance of communication among different nodes.
- The greedy forwarding mechanism is expressed in the form of probability; that is, each neighbor node is given a weight between 0 and 1 according to the distance, and the weight represents the probability that the node is selected as the next-hop node. In this paper, the nearby region around the current forwarder is divided into four parts according to the distance, and different weights are set to the nodes in different parts.

The rest of this article is organized as follows. Section 2 reviews related work. The network model and energy model are introduced in Section 3. Section 4 describes the design of THECB. Section 5 presents the analysis of THECB. The simulation performance results are introduced and discussed in Section 6. Finally, Section 7 concludes this article.

## 2. Related Work

### 2.1. Data Transmission of SIL-IoTs

As shown in Table 1, only a few studies have investigated the data transmission of the SIL-IoTs. The study of Lam et al. [7], which contributed to establishing a real-time pests monitoring system, is the only research considering data transmission between nodes in the mesh network. Besides, Qiu et al. [8] sent the data to the node with the radar sensor in one hop through a star topology-based wireless network. However, neither studies consider the communication energy management and characteristics of SIL-IoTs.

### 2.2. Two-Hop Neighbors Information Based Routing Algorithms

In the past decade, various geographic routing approaches in WSNs have been presented. Most of them focus on geographic forwarding based on 1-hop neighbors’ information. However, it has been proved that the routing algorithm based on 2-hop neighbors’ information increases the success rate of guaranteed delivery and leads to high-quality paths compared with 1-hop case [4].

#### 2.2.1. Related Works of Other Teams

Table 2 shows some classical routing algorithms of other teams utilizing the 2-hop neighbors’ information. TIE-GeR [9] and TN-CMAD [10] make routing decisions based on the distance from neighbors to the sink. The number of the neighbors of the current forwarder and its 1-hop neighbors is taken into account by T-GPSR [11] and EEPDBR [12] to select the next-hop nodes. Interestingly, in the algorithm proposed by Hu et al. [13], the forwarding node selection policy is according to the link quality determined by a new measure of minimum summation angle, for this algorithm is applied to a scenario where image sensor nodes need to transmit images to the mobile robot sinks. All the above algorithms are studied for the mobile Ad hoc networks, except TIE-GeR that does not specify the model, and T-GPSR that has the mobile sink but the fixed image sensor nodes. As to the network density, only Hu et al. emphasizes that the algorithm is designed for a sparse network. Besides, only TIE-GeR and EEPDBR consider the energy consumption, and none of the above algorithms discussed the interference in communication. All the above algorithms are designed for WSNs and belong to geographic routing.

#### 2.2.2. Related Works of Our Team

The duty-cycle scheme schedules sleeping and awake conditions for nodes to ensure long battery life. For instance, Han et al. proposed 2-hop neighbor information and a duty-cycle-based algorithm called TPGFplus [14]. After that, he also designed a cross-layer optimized routing scheme based on TPGFplus, which optimizes the physical layer, duty-cycle layer, and the routing layer to save energy [4]. Not surprisingly, the duty-cycle scheme leads to longer battery life but requires that other nodes can still form links when partial nodes sleep. However, due to the sparsity deployment of nodes in SIL-IoTs, making partial nodes sleep may result in some nodes having no neighbors to choose as the next-hop nodes for data forwarding, which does not meet the requirements of full connection in SIL-IoTs. Therefore, the duty-cycle scheme is not suitable for SIL-IoTs. Unlike the above studies, the nodes in THECB do not consider the duty-cycle scheme and would always be awake.

TPGFPlus is designed according to TPGF [15], and they both are our team’s previous works. Table 3 shows the characteristics of TPGF, TPGFPlus and THECB. TPGF makes routing decisions only based on the distance (from the current forwarder to the sink and from 1-hop neighbors to the sink). THECB and one of the forwarding mechanisms in TPGFPlus that we mainly consider both adopt the following factors: (1) distance from 2-hop neighbors to the sink, and (2) the residual energy to make the routing decision. Nevertheless, the calculation methods of these two factors are different between THECB and TPGFPlus, and THECB considers more factors in the routing decision to save more energy consumption of communication and reduce the unpredictable electromagnetic interference on communication. In addition, the *routing-loop situation* and *path-circle situation* [15] (also explained in Section 4.3.2 and Section 4.3.3 in this paper) can appear in these three algorithms. Nevertheless, to solve these two situations, TPGF and TPGFPlus utilize the Step Back and Mark method and the Label Based Optimization method, respectively, different from the two methods proposed in THECB.

In SIL-IoTs, data transmission is affected by high voltage discharges. Thus it is critical for SIL-IoTs nodes to reduce the data forwarding traffic of the nodes whose discharge numbers are relatively higher than those of other nodes. This important feature is ignored in previous works of both other teams and our team. In addition, these algorithms do not take into account the characteristic of the different energy consumption of various discharge numbers among different nodes in SIL-IoTs, thus neglecting to improve the energy consumption balance of SIL-IoTs.

## 3. System Model

### 3.1. Network Model

Based on the deployment strategies of SIL-IoTs proposed in [16], the location of every node is fixed, and the internode distance varies from 30 m to 150 m. One sink node with sufficient energy in this network is deployed at the boundary area of the covered region. Each SIL-IoTs node knows its location through an internal GPS module and stores the sink node’s location in advance. The number of every SIL-IoTs node’s 1-hop neighbors is more than or equal to one. All SIL-IoTs nodes are assumed to transmit their data to the sink node. Therefore, in this paper, nodes are sparsely deployed but fully connected in SIL-IoTs. There are many parameters used in this paper and Table 4 shows the meaning of important parameters.

### 3.2. Energy Model

#### 3.2.1. Transmission Energy Consumption Model

Like WSNs, every node in SIL-IoTs is responsible for transmitting its data collected through the equipped sensor modules to the sink. This communication process would cost a lot of energy. We adopt the following model [12] to calculate the energy consumption used for a node sending one bit of data to its neighbor:

(1) $$e_{send}=\left\{\begin{array}{cc} E_{cir}+\varepsilon_{fs}\cdot d^{2}, & d<d_{0}\\ E_{cir}+\varepsilon_{amp}\cdot d^{4}, & d\geq d_{0} \end{array}\right.$$

where $E_{cir}$ is assumed as the energy consumed by the transmitting circuit when transmitting a bit, and $\varepsilon_{fs}$ is defined as the energy consumption of the transmitting amplifier to send one bit of data in free space, and $\varepsilon_{amp}$ is similar to $\varepsilon_{fs}$ in the multipath fading channel. *d* represents the distance between the sender and receiver, and $d_{0}$ is a reference distance: when $d<d_{0}$, the channel model is a free space model. Otherwise, it is a multipath fading model.

The energy consumed by a node receiving one bit of data can be calculated as follow:

(2) $$e_{receive}=E_{cir}$$

#### 3.2.2. Energy Consumption Model of Keeping the Lure Lamp on

The SIL attracts migratory pests via using a lure lamp. The rated power of the lure lamp is 12 W, which is represented by $P_{L}$. Consequently, the energy consumption of the lure lamp is defined as:

(3) $$E_{L}=\int P_{L}\cdot t$$

where *t* denotes the working time of the lure lamp.

#### 3.2.3. Energy Consumption Model of Releasing High Voltage Pulse Currents

The SIL kills pests by releasing high voltage pulse currents. To the best of our knowledge, no previous research investigates the energy consumption of releasing high voltage discharges while pests contact with the metal mesh. In our previous work [17], to explore the quantitative index of discharge intensity, a discharge simulation module was adopted to simulate the discharge of the SIL when pests contact the metal mesh. Based on the equipment of our previous work [17], we designed the following experiment to obtain the approximate energy consumption of one discharge.

**Figure 2:**
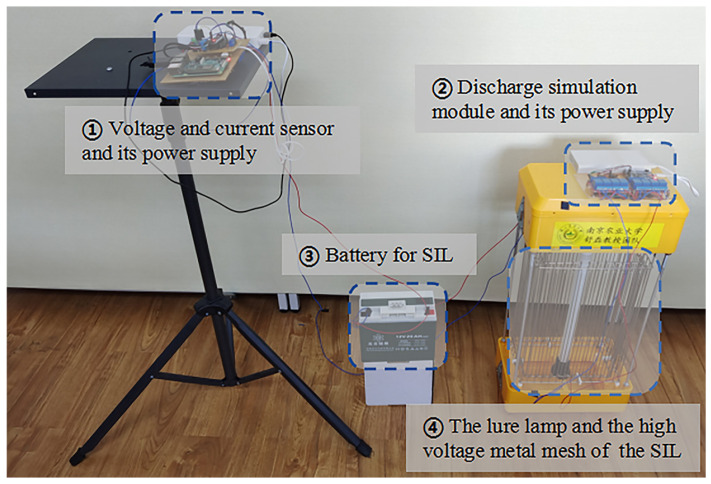
The experiment devices which are used to calculate energy consumption.

The experiment is carried out indoors to reduce the impact of environmental factors on the results. All devices are shown in Figure 2:

(1) The voltage and current sensor module are used to acquire data (10 times per second) for calculating energy consumption. It consists of a sensor (INA219) for acquiring circuit and voltage value, and a Raspberry Pi for storing data. This module is powered by Xiaomi battery (20,000 mAh);
(2) Compared with the actual discharge times, which are difficult to count when the SIL is killing pests, the discharge simulation module helps record the discharge times more accurately. The discharge simulation module includes eight electromagnetic relays (DC-5V), and one microprocessor (STC89C52RC). This module is designed to control and simulate different discharge frequencies using a program set up in the microprocessor. Its power supply is Xiaomi battery (20,000 mAh);
(3) The SIL is powered by a battery (20 AH) and not equipped with an unnecessary solar panel for our experiment;
(4) The lure lamp attracts pests, and the high voltage metal mesh can kill pests by releasing high voltage pulse currents.

When the SIL only turns on the lure lamp, the state is denoted as SATE1, and the state when the SIL turns on both the lure lamp and the discharge simulation module is represented as SATE2.

Voltage, current, and working time are collected under SATE1 and SATE2. When the voltage changes from U1 to U2, the energy consumption of the SIL in SATE1 and SATE2 are presented as $E_{SATE1}$, and $E_{SATE2}$. Besides, the working time in SATE1 and SATE2 are T1, and T2. The above parameters are characterized as follows:

(4) $$\begin{array}{cc} \; & E_{SATE1}=\int_{T1}P_{L}\cdot tdt\\ \; & E_{SATE2}=\int_{T2}UItdt\\ \; & E_{L}=\int_{T2}P_{L}tdt \end{array}$$

where *U* and *I* are the value of the voltage and current, the current of the SIL changes when SIL generates discharges. The time of releasing one high voltage discharge is especially short, and the voltage and current sensor modules do not have a high enough sampling frequency to acquire enough data over some time. Thus using integral to calculate $E_{SATE2}$ is not appropriate, for it can produce a great number of errors. To solve this problem, we assume that for the same battery, when the voltage changes from U1 to U2, the total energy consumption of the SIL in SATE1 can be approximately equal to that in SATE2, that is, $E_{SATE1}=E_{SATE2}$. Therefore, the energy consumed by one discharge of the SIL can be calculated through the following formula:

(5) $$E_{D}=E_{SATE2}-E_{L}=E_{SATE1}-E_{L}$$

**Figure 3:**
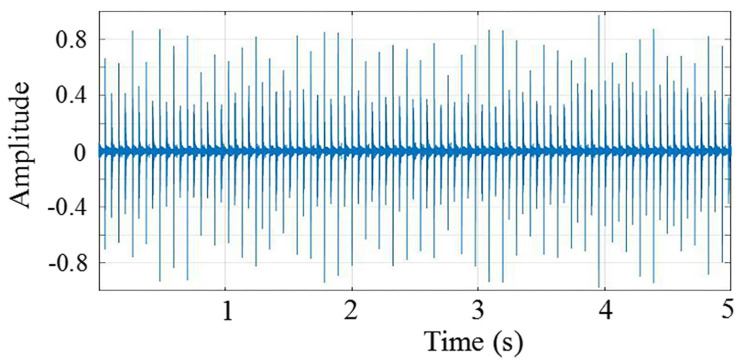
When the discharge simulation module controls the SIL to generate a discharge, it makes an obvious sound. A significant increase inordinate data means a discharge generation of the SIL.

Unlike our previous work, we found a fixed deviation between the actual discharge frequency and the set discharge frequency. To obtain the discharge times accurately, additional information is required. In the experiment, we found that when the discharge simulation module controls the SIL to generate a discharge, it makes an obvious sound. The sound data of the discharge simulation module can be utilized to acquire actual discharge frequency. Figure 3 describes the sound data of the discharge simulation module over some time after normalizing the amplitude. Therefore, a significant increase inordinate data of Figure 3 represents a discharge generation of the SIL. Hence, we obtain the approximate discharge frequency (0.1084357 seconds per discharge) through collecting and analyzing the sound data of the discharge simulation module when working.

In this way, the number of discharges is T2/0.1084357 for SATE2, and the average energy consumption of each discharge is about: $E_{D}=0.7$ J.

## 4. Proposed Algorithm

This section presents in detail the proposed THECB algorithm for SIL-IoTs. First, the architecture of THECB is briefly introduced, and then how to select the next-hop node, solve the *routing-loop situation* and the *path-circle situation* are introduced. Finally, the data packet is transmitted based on the established routing table.

### 4.1. THECB Framework

**Figure 4:**
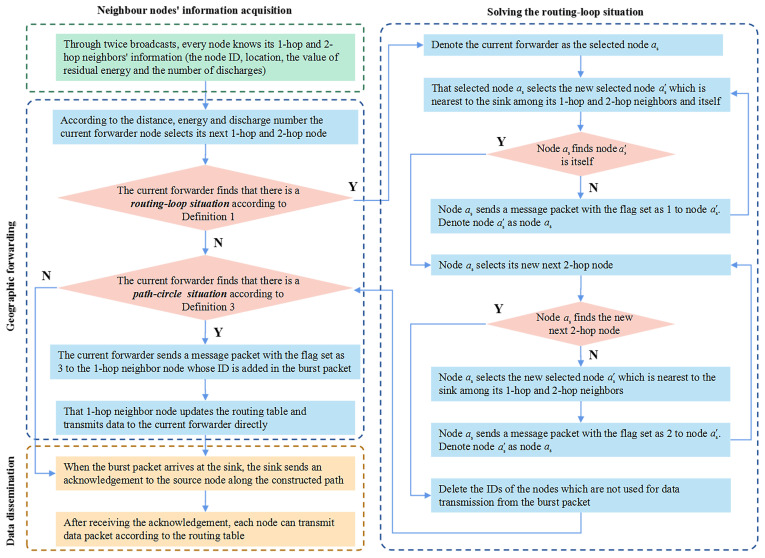
The flow chart of THECB.

The algorithm is divided into 3 phases and its flow chart is shown in Figure 4:

- **Neighbour nodes’ information acquisition:** All the nodes obtain their 1-hop and 2-hop neighbors’ IDs, locations, residual energy value, and the number of discharges through twice broadcasts.
- **Geographic forwarding:** The current forwarder considers the distance, residual energy, and the number of discharges to choose its next 1-hop and 2-hop nodes. Then, the current forwarder sends a burst packet to the next 1-hop and 2-hop nodes. These nodes add their IDs to this packet successively. During the exploring of a path, every node will solve the *routing-loop situation* and *path-circle situation* if these problems occur. Finally, each node obtains a routing table for maintaining the next 1-hop and 2-hop nodes.
- **Data dissemination:** When the burst packet arrives at the sink, the sink sends an acknowledgment to the source node along the constructed path. After receiving the acknowledgment, the nodes transmit data packets according to the routing tables. For any node, if the difference of related parameters (i.e., the value of residual energy and number of discharges) exceeds the threshold, the 1-hop, 2-hop neighbors and themselves would rebuild their routing tables.

**Figure 5:**
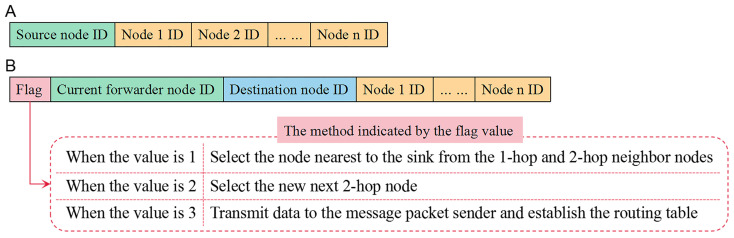
(**A**) The format of the burst packet. (**B**) The format of the message packet and the method indicated by each flag value.

Our scheme has three kinds of packets when building the routing table:

- **Beacon packet:** It is used to exchange information among neighbors during the twice broadcasts.
- **Burst packet:** It helps to explore the path. Its format is illustrated in Figure 5A. The ID of every node on the found path is sorted in this packet.
- **Message packet:** When solving the *routing-loop situation* and *path-circle situation*, this packet is adopted to inform nodes to choose the appropriate method. As shown in Figure 5B, this packet contains a flag indicating which method to choose.

### 4.2. Neighbor Nodes’ Information Acquisition

The following three steps are processed to obtain the information of 1-hop and 2-hop neighbor nodes:

Step 1: Every node broadcasts a beacon packet (containing the ID, location, residual energy value, and the number of discharges) to its neighbors at the maximum transmission power.

Step 2: Once the node receives the information, it adds its 1-hop neighbors’ information to the beacon packet.

Step 3: Every node broadcasts this new beacon packet. Finally, every node knows its 1-hop and 2-hop neighbors’ information.

### 4.3. Geographic Forwarding

#### 4.3.1. Select The Next 1-hop and 2-hop Node

**Figure 6:**
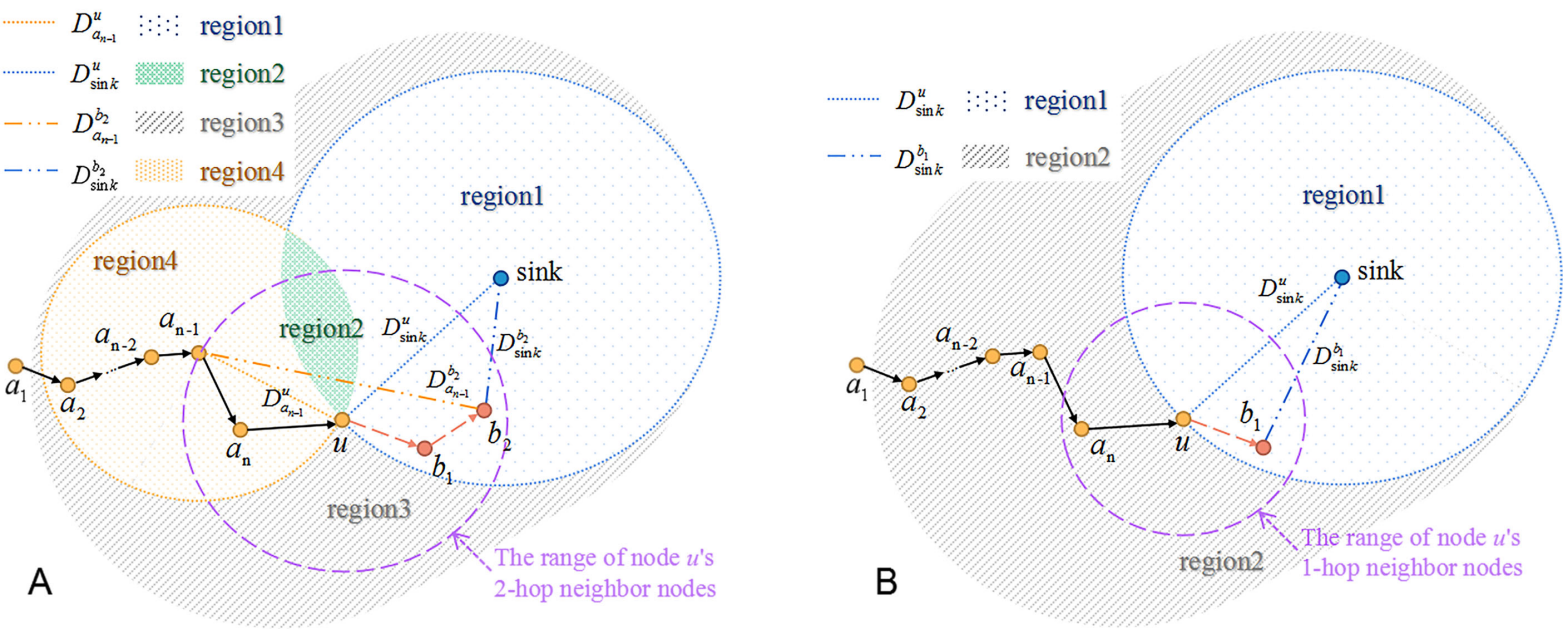
The greedy forwarding mechanism is expressed in the form of probability. (**A**) The greedy forwarding mechanism of THECB: According to the distance, the nearby region around the current forwarder *u* is divided into four parts: region1, region2, region3, and region4. The specific division rule is expressed in Equation (6). $b_{2}$ is the node that may be selected as the next 2-hop node by *u*. The distance from $b_{2}$ to the sink ($D_{sink}^{b_{2}}$) is shorter than the distance from *u* to the sink ($D_{sink}^{u}$); The distance from $b_{2}$ to $a_{n-1}$ ($D_{a_{n-1}}^{b_{2}}$) is longer than the distance from *u* to $a_{n-1}$ ($D_{a_{n-1}}^{u}$), which means $b_{2}$ is in region1, and is nearer to the sink and farther to the upstream forwarders than *u*. (**B**) The traditional greedy forwarding mechanism: The nearby region around the current forwarder *u* is divided into two parts. The neighbour node in region1 is closer to the sink than the current forwarder, while the neighbour node in region 2 is farther to the sink than the current forwarder.

As shown in Figure 6A, for the current forwarder *u*, node $a_{n-1}$, $a_{n}$, $b_{1}$ and $b_{2}$ are represented as its last 2-hop, last 1-hop, next 2-hop and next 1-hop nodes, respectively. $D_{a_{n-1}}^{u}$, $D_{sink}^{u}$, $D_{a_{n-1}}^{b_{2}}$ and $D_{sink}^{b_{2}}$ denote the distance from node $a_{n-1}$ to *u*, the sink to *u*, $a_{n-1}$ to $b_{2}$ and the sink to $b_{2}$, respectively. The nearby region around node *u* is divided into four parts, and weight $\beta_{i}$ is set for *i*th region (*i* = 1, 2, 3, 4). The division rule is shown as following:

(6) $$\left\{\begin{array}{cc} D_{sink}^{b_{2}}<D_{sink}^{u}\;\&\;D_{a_{n-1}}^{b_{2}}>D_{a_{n-1}}^{u}, & i=1\\ D_{sink}^{b_{2}}<D_{sink}^{u}\;\&\;D_{a_{n-1}}^{b_{2}}<D_{a_{n-1}}^{u}, & i=2\\ D_{sink}^{b_{2}}>D_{sink}^{u}\;\&\;D_{a_{n-1}}^{b_{2}}>D_{a_{n-1}}^{u}, & i=3\\ D_{sink}^{b_{2}}>D_{sink}^{u}\;\&\;D_{a_{n-1}}^{b_{2}}<D_{a_{n-1}}^{u}, & i=4 \end{array}\right.$$

The shorter distance from the chosen next-hop node to the sink is set as the prime target, and the long-distance from the chosen next-hop node to node $a_{n-1}$ is set as the secondary target. Intuitively, nodes in region1 are the best choice of next hop nodes because they are farther to node $a_{n-1}$ but nearer to the sink than node *u*. Nodes in region2 transmit data closer to upstream forwarders, and thus they are the second choice. Nodes of region3 or region4 would transmit data packets farther away from the sink node than node *u*, so these nodes are the worse choices than those of region1 or region2.

Through the above analysis, the weight factors of these four regions could be set as: $\beta_{1}<\beta_{2}<\beta_{3}<\beta_{4}$, which indicates that a smaller weight represents a better choice of next-hop nodes.

In summary, the greedy forwarding mechanism is expressed in the form of probability, that is, each neighbor node is given a weight between 0 and 1 according to the distance, and the weight represents the probability that the node is selected as the next-hop node. The traditional greedy forwarding mechanism (the current forwarder chooses the neighbor node that is closer to the sink than itself) can also be converted into the following probability forms according to the above idea: The weight of the neighbor node that is closer to the sink than the current forwarder is set as 1, which means that neighbor node is the better choice. The following formula can express the above traditional greedy forwarding mechanism:

(7) $$\left\{\begin{array}{cc} D_{sink}^{b_{1}}<D_{sink}^{u}, & i=1\\ D_{sink}^{b_{1}}>D_{sink}^{u}, & i=2 \end{array}\right.$$

Therefore, as depicted in Figure 6B, the nearby region around the current forwarder *u* is divided into two parts. The neighbor node in region 1 is closer to the sink than the current forwarder, while the neighbor node in region 2 is farther to the sink than the current forwarder. Thus, the weight factor of region 1 and region 2 could be set as 1 and 0, respectively.

According to the number of discharges, nodes are graded into three discharge levels (DL): DL1 (the discharge number is relatively the smallest), DL2 (the discharge number is relatively medium), and DL3 (the discharge number is relatively the highest). The weights of them are: $\mu_{1}$, $\mu_{2}$, $\mu_{3}$, where $\mu_{1}<\mu_{2}<\mu_{3}$ and $\mu_{1}+\mu_{2}+\mu_{3}=1$. Assume that $\phi =\mu^{b_{1}}+\mu^{b_{2}}$, where $\mu^{b_{1}}$ and $\mu^{b_{2}}$ represent the weight factor μ of node $b_{1}$ and $b_{2}$, respectively. It is critical to reduce the data forwarding traffic of nodes whose discharge numbers are relatively higher than those of other nodes because high voltage discharges have interference on communication [5,6]. Clearly, smaller ϕ indicates less interference on communication.

**Figure 7:**
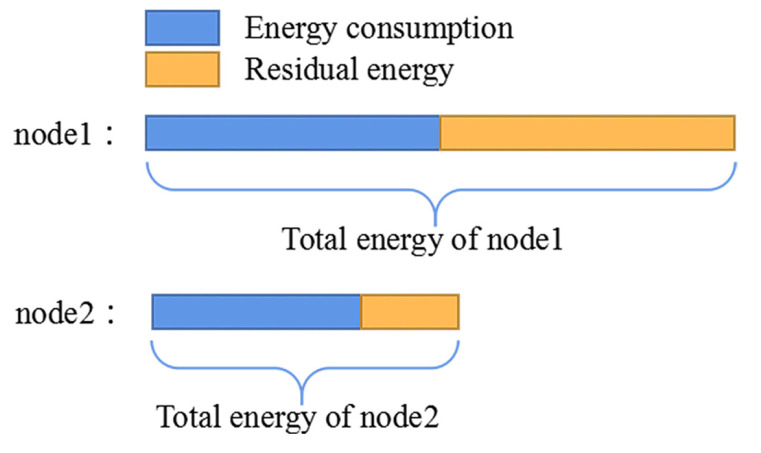
The energy consumption of node2 is less than that of node1. However, node1 consumes only 50% of its total energy while node2 consumes about 80% of the whole energy.

Finally, *P* is defined as the parameter to select the next 1-hop and 2-hop nodes:

(8) $$P=\phi \times \beta \times (\alpha D_{sink}^{b_{2}}+\left(1-\alpha \right)E)$$

where α is a weight factor between 0 and 1 that determines the relative significance placed on $D_{thesink}^{b_{2}}$ and *E*. *E* denotes the energy consumption factor, which is calculated as:

(9) $$E=\frac{e_{u}}{E_{res\_u}}+\frac{e_{b_{1}}}{E_{res\_b_{1}}}+\frac{e_{b_{2}}}{E_{res\_b_{2}}}$$

where $e_{u}$, $e_{b_{1}}$ and $e_{b_{2}}$ represent the energy consumption of communication by *u*, $b_{1}$ and $b_{2}$, respectively. $E_{res\_u}$, $E_{res\_b_{1}}$ and $E_{res\_b_{2}}$ denote the residual energy of *u*, $b_{1}$ and $b_{2}$, which is defined as:

(10) $$E_{res}=E_{ini}-E_{lamp}-E_{ele}$$

where $E_{ini}$ denotes the initial energy. $E_{lamp}$ and $E_{ele}$ represent the energy consumption of the lure lamp and discharges, respectively. As shown in the example of Figure 7, the energy consumption of node2 is less than that of node1, but accounts for about 80% of the whole energy of node2. However, node1 consumes about only 50% of its total energy. Obviously, node1 is the better choice. Therefore, we adopt the ratio of communication energy consumption ($e_{u}$, $e_{b_{1}}$ and $e_{b_{2}}$) to residual energy ($E_{res\_u}$, $E_{res\_b_{1}}$ and $E_{res\_b_{2}}$) to evaluate the energy consumption of nodes.

In summary, the current forwarder *u* calculates *P* of all optional paths and selects the path with the smallest *P*, since the smallest ϕ, β, $D_{sink}^{b_{2}}$ and *E* indicate the best choice of the alternative paths. Especially, due to the different magnitude of $D_{thesink}^{b_{2}}$ and *E*, it is necessary to normalize them respectively.

Finally, the current forwarder *u* sends a burst packet to the next 1-hop and 2-hop nodes. These nodes add their IDs to this packet successively.

#### 4.3.2. Solve Routing-Loop Situation

**Figure 8:**
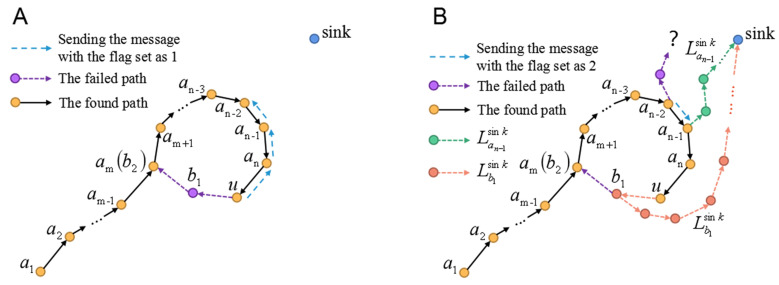
(**A**) The current forwarder *u* finds that if it chooses node $b_{2}$ as the next 2-hop node (as shown by the purple dotted line), there will be a routing loop $\underset{a_{m}a_{m+1}\cdots a_{n}ub_{1}a_{m}\left(b_{2}\right)}{\to }$. Then, *u* selects the node $a_{n-1}$ with the smallest distance to the sink from its neighbors $a_{n-1}$, $a_{n}$, *u*, $b_{1}$, and $b_{2}$. After that, as shown by the blue dotted line, *u* sends a message packet to $a_{n-1}$ to inform $a_{n-1}$ to select the node nearest to the sink from the neighbors. In this way, we can acquire the node $a_{n-2}$ which is nearest to the sink on the found path. (**B**) If $a_{n-2}$ could not find any new next 2-hop node to break the loop, $a_{n-2}$ will send the message packet with the flag set as 2 to $a_{n-1}$ (as shown by the blue dotted line). $a_{n-1}$ is nearest to the sink among the neighbors of $a_{n-2}$. Then, $a_{n-1}$ selects the new next 2-hop node. The green dotted line denotes the new path explored by $a_{n-1}$.

**Definition** **1** **(Routing-Loop Situation).***Let $A=\vec{a_{1}\cdots a_{m-1}a_{m}a_{m+1}\cdots a_{n}u}$ denote all the nodes on the found path from node $a_{1}$ to u, e.g., Figure 8A. If the next 1-hop or 2-hop node selected by the current forwarder u belongs to A, the data packet will be circularly transmitted among the previous forwarders $A_{Loop}=\vec{a_{m}a_{m+1}\cdots a_{n}ub_{1}a_{m}\left(b_{2}\right)}$, and this kind of situation is defined as a*routing-loop situation*, as depicted in Figure 8A.*

**Definition** **2** **(Block Circle and Block Line).***In Definition 1, if $a_{m}$ has only three 1-hop neighbors ($a_{m-1}$, $a_{m+1}$, $b_{1}$), this situation is defined as a* block circle *. As shown in Figure 8A, if the node $a_{m}$ at the end of the path $\vec{a_{1}\cdots a_{m-2}a_{m-1}a_{m}}$ has only one neighbor ($a_{m-1}$) and the other nodes have only two neighbors, we define this situation as a* block line. *These cases also make the data transmitted among the previous forwarders because the next hop nodes can only be chosen from the previous forwarders. Therefore, the* block circle *and* block line *are the special cases of the* routing-loop situation.

The *routing-loop situation* may also appear in our algorithm. The *routing-loop situation* may also appear in our algorithm. The following approach is proposed to solve this situation.

Step 1: If the current forwarder *u* finds that the ID of its next 2-hop node has been stored in the burst packet which has the IDs of all nodes on the found path (mentioned in Section 4.1), there occurs a *routing-loop situation* according to Definition 1. Then, *u* is denoted as the selected node $a_{s}$.

Step 2: That selected node $a_{s}$ selects the new selected node $a_{s}^{{}^{\prime }}$ which is nearest to the sink among itself and its 1-hop and 2-hop neighbors in *A* (e.g., in Figure 8A, *u* calculates the distances from $a_{n-1}$, $a_{n}$, *u*, $b_{1}$, and $b_{2}$ to the sink, respectively, and selects the node $a_{n-1}$ with the smallest distance).

Step 3: If $a_{s}$ finds that $a_{s}^{{}^{\prime }}$ is itself, that is, there are no neighbors on the found path *A* nearer to the sink than itself, Step 4 will be executed. Otherwise, node $a_{s}$ sends node $a_{s}^{{}^{\prime }}$ a message packet with the flag set as 1. Denote node $a_{s}^{{}^{\prime }}$ as node $a_{s}$, and then execute Step 2. The above Step 2 and Step 3 are repeated until $a_{s}$ is the nearest node to the sink among the nodes on the loop. (e.g., In Figure 8A, node $a_{n-2}$ has the smallest distance to the sink than other nodes in *A* and it is denoted as the final $a_{s}$).

Step 4: Node $a_{s}$ (e.g., $a_{n-2}$) selects its new next 2-hop node from the nodes ⊈A.

Step 5: If $a_{s}$ does not find a new path to break the loop, it selects the new selected node $a_{s}^{{}^{\prime }}$ (e.g., $a_{n-1}$) which is nearest to the sink among its 1-hop and 2-hop neighbors in *A*. Then, $a_{s}$ sends a message packet with the flag set as 2 to node $a_{s}^{{}^{\prime }}$. Finally, denote node $a_{s}^{{}^{\prime }}$ as node $a_{s}$, and then execute Step 4. Therefore, Step 4 and Step 5 are executed repeatedly until a new path is built to break the loop (e.g., in Figure 8B, if we assume that $a_{n-2}$ could not find that new path for some reason, node $a_{n-1}$ selects the new next 2-hop node to build that path).

In summary, starting from node $a_{s}$ (e.g., $a_{n-2}$), the nodes on the found path *A* find their new next 2-hop nodes in a specified order until a new path is built to break the loop.

Step 6: If a new path without ***routing-loop situation*** is built, delete the IDs of the nodes which are not used for data transmission from the burst packet (e.g., $b_{1}$, *u*, $a_{n}$ in Figure 8B).

**Figure 9:**
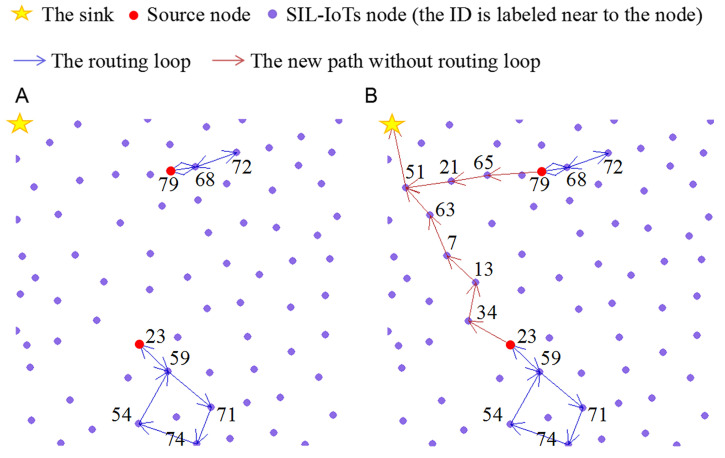
Two *routing-loop situations* occur in THECB during the simulation. (**A**) the routing loop: 79→68→72→68; 23→59→71→74→54→59. (**B**) the new path without loop: 79→65→21→51; 23→34→13→7→63→51.

Figure 9 shows two *routing-loop situations* occurring in THECB during the simulation. The ID is labeled near the node. To solve this problem, the node (ID 23, ID 79) nearest the sink among all nodes on the found path explores the new path.

The Step Back and Mark method proposed by [15] is designed to solve the *block line* (in [15], the *block line* has only one node and is named as the *block node*). In this method, if the current forwarder has no 1-hop neighbors except its previous forwarders, which means the next-hop node can only be chosen from the previous forwarders, it will step back to its last 1-hop node which will try to find a new available next-hop node. This step will be executed repeatedly until the new available next-hop node is found. Therefore, based on this mechanism, the Step Back and Mark (SBM) method can be extended to solve the *routing-loop situation* which is more general than the *block line*. The new mechanism is the following: If the next-hop node is chosen from the previous forwarders, the next-hop node will step back to its last 1-hop node which will try to find a new available next-hop node. This step will be executed repeatedly until the new available next-hop node is found. This method can be summarized as: In the order from the node that finds this situation to the source node, nodes on the built path attempt to find a newly available path (e.g., in Figure 8B, nodes explore the new path in the following order: $b_{1}\to u\to a_{n}\to a_{n-1}\to \cdots$).

In our method, the nodes explore the new path according to the sort of distances from these nodes to the sink. As shown in Figure 8B, let $L_{b_{1}}^{sink}$ and $L_{a_{n-1}}^{sink}$ denote the new path found by $b_{1}$ using the SBM method, and found by $a_{n-1}$ based on our method, respectively. It is seen that $L_{b_{1}}^{sink}$ is longer than $L_{a_{n-1}}^{sink}$ because $a_{n-1}$ is closer to the sink than $b_{1}$. In particular, the difference between the two paths would be larger in more sparse networks. Although sending message packets in our method when solving the *routing-loop situation* costs some energy, due to (1) this process is only executed during building the routing table and may not consume energy frequently, and (2) the new path to break the loop established by our method may be shorter than that of the SBM method, especially in the sparse network such as SIL-IoTs, which indicates our method may save more energy consumption of communication and improve the transmission speed than the Step Back and Mark method in multiple data transmission after the routing table is established, it is still highly significant to design and adapt our method.

#### 4.3.3. Solve Path-Circle Situation

**Figure 10:**
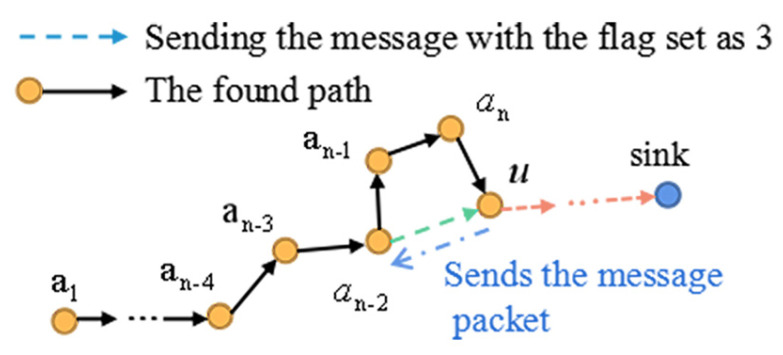
The current forwarder *u* finds that the ID of its 1-hop neighbor node $a_{n-2}$ is in the burst packet, which means a *path-circle situation* occurs in the found path. Then, node *u* informs node $a_{n-2}$ to transmit data to *u* directly through sending a message packet with the flag set as 3. Finally, node $a_{n-2}$ updates the routing table.

**Definition** **3** **(Path-Circle Situation).** *Let A denote all the nodes on the found path from node $a_{1}$ to u. For a node u∈A, if more than one node of A is node u’s neighbors, as shown in Figure 10, we consider that there is a path-circle situation.*

Although THECB is based on 1-hop and 2-hop neighbors’ information, *path-circle situation* may still occur. Solving this situation means optimizing the found path with the least number of nodes, so the following optimization method is proposed:

Because the ID of every node on the found path is stored in the burst packet (mentioned in Section 4.1), the current forwarder *u* knows all the upstream nodes’ IDs. Therefore, if *u* finds its 1-hop neighbor node *a* belongs to *A* (except last 1-hop and 2-hop nodes of *u*), which means there is a *path-circle situation* in the found path, node *u* sends a message packet with the flag set as 3 to node *a*. Then, node *a* updates the routing table and transmits data to *u* directly. Obviously, *path-circle situation* is solved during exploring the path in our method, while in the Label Based Optimization method used in TPGF [15] and TPGFPlus [4,14] the solution of that situation depends on sending back an acknowledge from the sink to the source node after the whole path is built.

**Figure 11:**
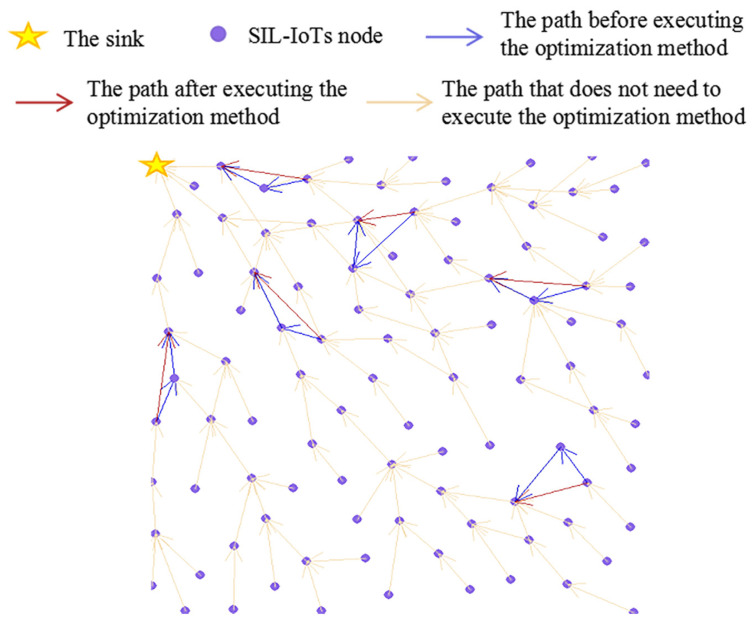
During the simulation of THECB, some *path-circle situations* appear. After executing the optimization method, paths have the least number of nodes.

Figure 11 depicts some *path-circle situations* occurring in THECB and the results after executing the optimization method during the simulation.

### 4.4. Data Dissemination

When the burst packet arrives at the sink, the sink sends an acknowledgment to the source node along the built path. After receiving the acknowledgment, each node can transmit the data packet according to the routing table.

Suppose the node finds that the change of its residual energy or number of discharges exceeds the threshold, or its residual energy is lower than the energy threshold that ensures communication. In that case, it will broadcast the beacon packet. Then the neighbors receiving this packet add their 1-hop neighbors’ information to the beacon packet and broadcast the new beacon packet again. All the nodes receiving the beacon packet resend the burst packet to establish a new routing table. The neighbors whose residual energy is lower than the threshold will not be used to relay data packet.

## 5. Analysis

**Theorem** **1.** *For a sink node, no matter where it locates in the network, all the other nodes can find a path to transmit their data using the THECB method.*

**Figure 12:**
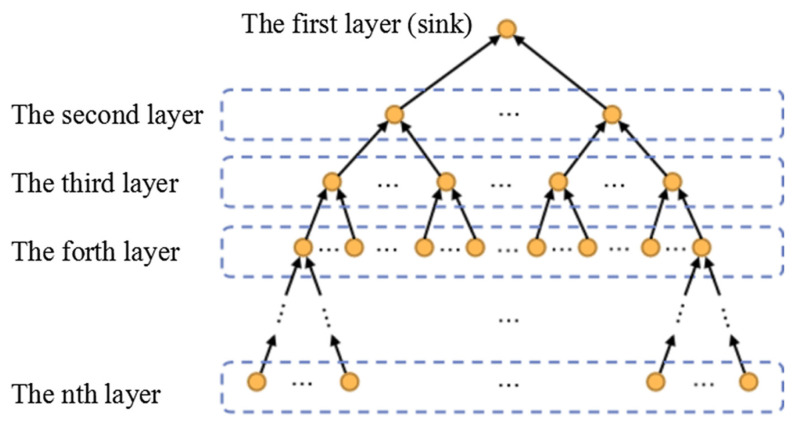
DEDST: THECB can convert the WSNs to a Distance, Energy, and Discharge level Search Tree (DEDST). The first layer of the tree is the sink node. The last *n*-hop nodes of the sink are classified into the *n*th layer.

**Proof.**  Based on the idea of [15], THECB can convert the WSNs to a Distance, Energy, and Discharge level Search Tree (DEDST), e.g., Figure 12. The first layer of the tree is the sink node, namely, the root node of the tree. The second layer is the last 1-hop node of the sink node, and the third layer is the last 2-hop node of the sink node. In this way, each node can be classified into DEDST. Hence, the last *n*-hop nodes of the sink can be classified into the *n*th layer of DEDST. □

## 6. Performance Evaluation

In this section, we evaluate the performance of THECB via simulation experiments. We present our simulation environments and performance metrics and evaluate the performance results. Moreover, the comparison between THECB and TPGFPlus, which ignores the requirement of the node-disjoint path, is invested.

### 6.1. Simulation Setup

We evaluate the performance of THECB through the sensor network simulator NetTopo [18]. Table 5 shows the value of important parameters. The number of nodes randomly distributed in a 2D area of 800 m × 800 m increases from 100 to 250, determining the network density increase. The internode distance is more than 30 m.

Each SIL-IoTs node contains a variety of sensor modules to obtain data; thus, each node is set to generate a packet with the size of 50kb and transmit it to the sink every 5 min. We evaluate the performance of nodes after working for half an hour. The sink is deployed at the fixed location of (200 m, 200 m), which receives data from all nodes.

The energy consumption of sending and receiving data for each node is based on the first radio model, which is mentioned in Section 3.2.1. The maximum transmission radius Tr of each node is changed from 90 m to 120 m (each time increased by 10 m).

This paper mainly studies the performance of THECB when the SIL turns on the lure lamp and kills pests by generating discharges at night. Therefore, we set the initial energy $E_{ini}$ as the energy of the battery charged by the solar panel in the daytime. The working time of the lure lamp at night is a fixed value set before, and the number of discharges can be estimated according to historical data.

**Figure 13:**
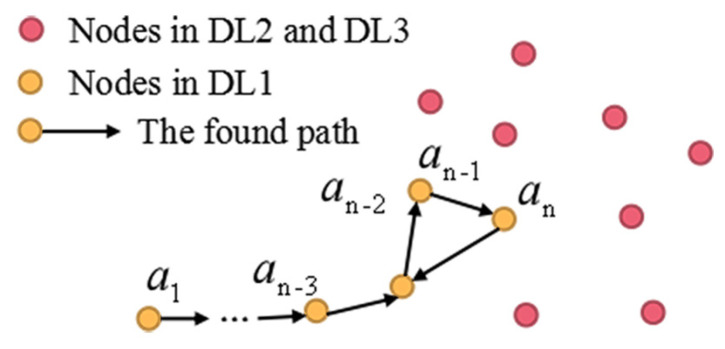
If the value of $\mu_{1}$ is quite small and the value of $\mu_{3}$ is quite big, the current forwarder prefers to select the node in discharge level DL1 as the next hop node. When almost all the neighbors except the last hop nodes of the current forwarder are in discharge levels DL2 and DL3, the current forwarder will choose its next hop node from the previous forwarders, which causes the appearance of the *routing-loop situation*.

According to the experimental data, the number of discharges in DL1, DL2, and DL3 (mentioned in the Section 4.3) are assumed to change from 300 to 1500, 1500 to 2250, and 2250 to 3000. Through many simulations, we find that if the difference among $\mu_{1}$, $\mu_{2}$, and $\mu_{3}$ is set relatively large (e.g., the value of $\mu_{1}$ is quite small, or the value of $\mu_{3}$ is quite big), that is, few nodes in DL3 or DL2 are selected as the next hop nodes, the paths will become quite long and the *routing-loop situation* can easily occur, as depicted in Figure 13. Therefore, the weights of DL1, DL2, and DL3 can be set as: $\mu_{1}$ = 0.2, $\mu_{2}$ = 0.3, and $\mu_{3}$ = 0.5, respectively. However, it is also found that setting the difference among $\beta_{1}$, $\beta_{2}$, $\beta_{3}$ and $\beta_{4}$ to be large (e.g., $\beta_{1}$ and $\beta_{2}$ are set relatively small, or $\beta_{3}$ and $\beta_{4}$ are set relatively big) can increase the probability of selecting the nodes close to the sink as the next hop nodes, thereby reducing the appearance of the *routing-loop situation*. Based on the above simulation results, $\beta_{1}$, $\beta_{2}$, $\beta_{3}$ and $\beta_{4}$ is set as 0.05, 0.15, 0.3 and 0.5, respectively.

Twenty different network deployments are generated by using 20 different seeds. For a certain proportion of node numbers in DL1, DL2, and DL3, the discharge numbers of different nodes in each network deployment are randomly set 10 times. Thus, the average performance results are obtained from 200 different cases. The following three simulation scenarios are designed to illustrate the performance of THECB.

- **Proportion of different discharge levels scenario:** The ratio of node number in DL1, DL2 and DL3 is denoted as $N_{DL1}$, $N_{DL2}$, and $N_{DL3}$, which satisfy: $N_{DL1}+N_{DL2}+N_{DL3}=1$. This paper changes the proportion of node numbers in different discharge levels to simulate various numbers of discharges for the SIL nodes deployed in the farmland.
- **Network density scenario:** The network density, presented by ρ, is the mean number of neighbors per node and can be defined as $\rho =k\cdot \frac{\pi \cdot TR^{2}}{800\;\times \;800}-1$, where *k* denotes the total number of nodes in the 800 m × 800 m area [19]. ρ is positively related to the number of nodes under a certain TR. In this paper, we change the network density ρ by changing the nodes’ total number.
- **Transmission radius scenario:** The increase of data transmission distance leads to more energy consumption of nodes for sending data. Therefore, it is significant to invest the impact of increasing the TR to the performance of THECB when other conditions remain unchanged.

TPGFPlus [4] is a geographic routing algorithm that is also based on 1-hop and 2-hop neighbors’ information. As shown in Table 3, THECB and one of the forwarding mechanisms in TPGFPlus select next-hop nodes according to the following two factors: (1) distance from 2-hop neighbors to the sink, and (2) the value of residual energy to make the routing decision. However, the calculation methods of these two factors are different between THECB and TPGFPlus. THECB also considers the discharge number when making the routing decision to reduce the unpredictable electromagnetic interference in communication. In addition, the *routing-loop situation* and *path-circle situation* [15] (also explained in Section 4.3.2 and Section 4.3.3 in this paper) can appear in these two algorithms. However, THECB and TPGFPlus solve the two situations based on two different methods.

Because of the similarities and differences between THECB and TPGFPlus described in Table 3 and Section 2, we compare THECB with TPGFPlus to have in-depth performance analysis. Specially, we only consider the energy-aware forwarding mechanism of the three forwarding mechanisms in TPGFPlus. Besides, the requirement of the node-disjoint path in wireless multimedia sensor networks is ignored because we only investigate the routing decision method not the application scenario of TPGFPlus. In the following, TPGFPlus that ignores the node-disjoint path requirement is named TPGFPlus Ignores Node-Disjoint (TPGFPlus-IND).

### 6.2. Performance Metrics

We analyze the following performance metrics to evaluate our scheme.

#### 6.2.1. Total Energy Consumption Ratio

Total energy consumption ratio [20] is defined as $E_{rat}=\sum_{j=1}^{n}e_{j}\backslash E_{res}^{j}\;\left(j=1,2,\ldots ,n\right)$, where $e_{j}$ and $E_{res}^{j}$ represent the energy consumed by communication and the residual energy of node *j*, respectively. The number of the whole nodes is denoted as *n*. This metric is used to evaluate the energy consumption of communication.

#### 6.2.2. Variance of Energy Consumption Ratio

In statistics, variance is widely used to evaluate the discrete degree of data [21]. The variance of energy consumption ratio is used to evaluate the difference of energy consumption among different nodes, namely, the energy consumption balance among different nodes involved in communication. This metric can be expressed as: $E_{var}=\left(\sum_{j=1}^{n}{\left(e_{j}\backslash E_{res}^{j}-E_{ret}\backslash n\right)}^{2}\right)\backslash n\;\left(j=1,2,\ldots ,n\right)$.

#### 6.2.3. Data Forwarding Traffic

As mentioned in Section 4.3, high voltage discharges have interference in communication; thus, the data forwarding traffic of the nodes whose discharge numbers are relatively higher than those of other nodes should be reduced. Based on this principle, this metric indicates communication interference in the SIL-IoTs scenario. For the nodes in DL1, DL2 and DL3, we define the data forwarding traffic as $T_{DL1}$, $T_{DL2}$ and $T_{DL3}$, respectively.

### 6.3. Performance of THECB with Varied Proportion of Different Discharge Levels

In this subsection, we investigate the performance of the two algorithms in the following 15 cases. In each case, the values of $N_{DL1}$, $N_{DL2}$ and $N_{DL3}$ (mentioned in Section 6.1) are shown in Table 6. We fix the transmission radius as 100 m and the number of nodes as 160. In addition, we change α of Equation (8) from 0.5 to 1.0 (each time increased by 0.02) to choose an appropriate value of α for the following simulations.

**Figure 14:**
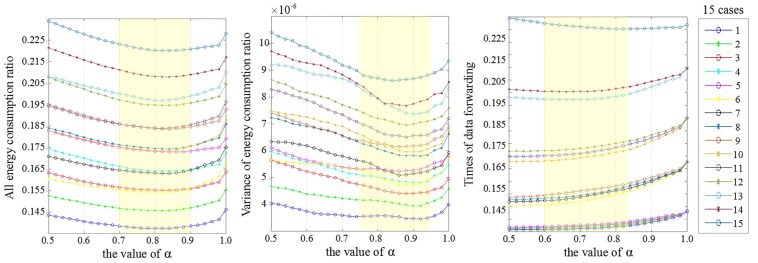
Under 15 cases, the results of $E_{rat}$, $E_{var}$ and $T_{DL3}$ are shown in this figure when α changes from 0.5 to 1.0. The yellow bar marks the lower value of metrics.

Figure 14 depicts $E_{rat}$, $E_{var}$ and $T_{DL3}$ when the value of α changes from 0.5 to 1.0. In each figure, a yellow bar marks the lower value of metrics. According to the results, we set the value of α to 0.8 in the following simulations.

**Figure 15:**
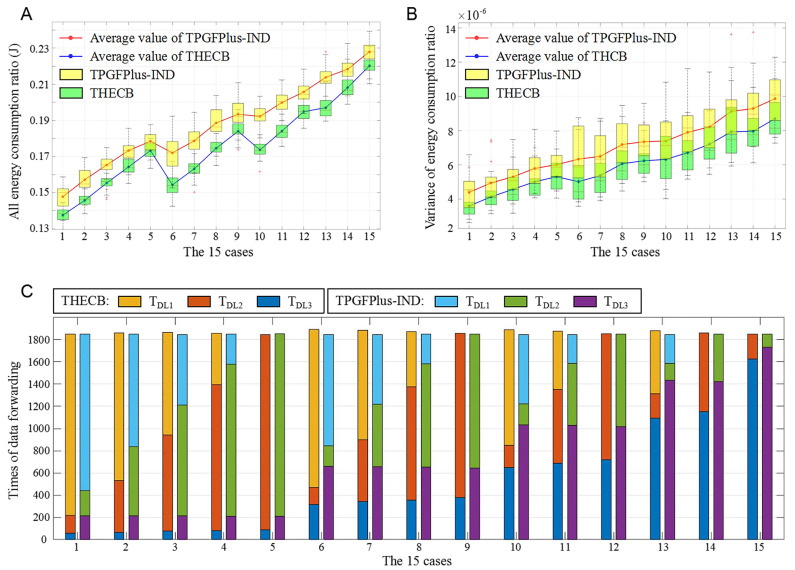
The performance of these algorithms with a varied proportion of different discharges levels. (**A**) Total energy consumption ratio. (**B**) Variance of energy consumption ratio. (**C**) The times of participating in data forwarding.

Figure 15A shows $E_{rat}$ of THECB and TPGFPlus-IND in the above 15 cases. It is easy to see that $E_{rat}$ of TPGFPlus-IND is approximately 0.01 to 0.02 higher than that of THECB in the above cases, which means that for the various discharge numbers of the SIL-IoTs nodes in the farmland, our algorithm always performs better in saving energy consumption of communication. In addition, the results illustrate that the growth of $E_{rat}$ is related to the values of $N_{DL1}$, $N_{DL2}$, and $N_{DL3}$. This phenomenon can be explained as follows: More discharge numbers lead to more energy consumption, making the denominator value of the energy consumption ratio smaller. Thus, the more nodes with high discharge numbers (the sum of $N_{DL2}$ and $N_{DL3}$) make the total energy consumption ratio greater. For example, in case1 to case5, $N_{DL3}$ remains unchanged, and $E_{rat}$ rises with the increase of $N_{DL2}$. In case 6, although $N_{DL3}$ increases, $E_{rat}$ suddenly diminishes since $N_{DL2}$ decreases more.

The trends of $E_{var}$ for both algorithms in Figure 15B are similar to $E_{rat}$ in Figure 15A. Besides, THECB achieves smaller $E_{var}$ than TPGFPlus-IND, which indicates that THECB has a better energy consumption balance of communication. Notably, the energy consumption balance of communication among nodes will become worse for both algorithms if the discharge numbers of most nodes become higher (the number of nodes in DL2 and DL3 becomes higher).

For the nodes of different discharge levels, $T_{DL1}$, $T_{DL2}$ and $T_{DL3}$ under all cases are shown in Figure 15C. The results indicate that the data forwarding traffic of all nodes ($T_{DL1}$ + $T_{DL2}$ + $T_{DL3}$) has no significant change in 15 cases. $T_{DL3}$ is always significantly smaller in THECB than that in TPGFPlus-IND. It benefits from additional parameter μ in THECB according to different discharge levels of nodes, making the node whose discharge number is relatively higher than those of other nodes difficult to be selected as the next-hop node. Compared to TPGFPlus-IND, $T_{DL2}$ is almost similar to that of THECB; however, $T_{DL1}$ is higher in THECB. Consequently, THECB prefers to utilize the nodes whose discharge numbers are relatively lower than those of other nodes to forward data, which means THECB has less unpredictable electromagnetic interference on communication than TPGFPlus-IND in the SIL-IoTs scenario.

### 6.4. Performance of THECB with Varied Network Densities

We fix the transmission radius as 100 m, and vary the number of nodes from 100 to 250 (each time increased by 30). Therefore, the network density is 3.91, 5.38, 6.85, 8.32, 9.79, and 10.28, respectively. The proportion of nodes in different discharge levels is set as: $N_{DL1}$:$N_{DL1}$:$N_{DL1}$ = 4:3:3.

**Figure 16:**
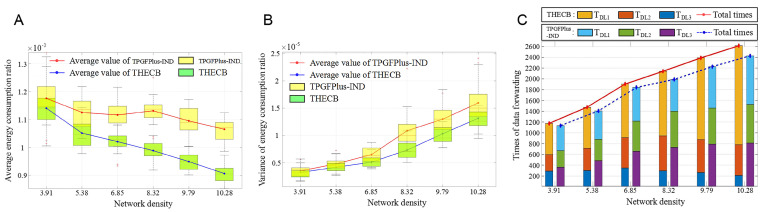
The performance of these algorithms with varied network densities. (**A**) Average energy consumption ratio. (**B**) Variance of energy consumption ratio. (**C**) The times of participating in data forwarding.

We use average $E_{rat}$, which is defined as $E_{rat}=\frac{1}{n}\sum_{j=1}^{n}e_{j}\backslash E_{res}^{j}\;\left(j=1,2,\cdots ,n\right)$, to evaluate the performance in saving energy consumption of communication. *n* is the number of nodes. As demonstrated in Figure 16A, average $E_{rat}$ becomes smaller as the network density increases, which indicates both algorithms perform better in saving energy consumption of communication with a higher network density. In particular, the average $E_{rat}$ of THECB shows a faster downward trend and can be reduced by 2.55%, 6.67%, 8.48%, 12.83%, 13.64%, and 15.42% compared with TPGFPlus-IND under different densities. The above results confirm that our algorithm, which utilizes energy consumption ratio instead of energy consumption, can significantly improve the performance in saving energy consumption of communication.

Figure 16B shows that there is little difference between the initial values of $E_{var}$ in THECB and TPGFPlus-IND. However, when the density reaches 10.28, the $E_{var}$ in THECB and TPGFPlus-IND have up to 1.25$\times 10^{-5}$ and 1.6$\times 10^{-5}$, respectively. It is seen that compared to TPGFPlus-IND, THECB achieves the smaller value and amplification of $E_{var}$. Therefore, THEE has a better energy consumption balance of communication and will have a higher advantage than TPGFPlus-IND if the density rises.

Figure 16C shows that the sum of $T_{DL1}$, $T_{DL2}$, and $T_{DL3}$ increases under a higher network density. Moreover, the values of $T_{DL1}$, $T_{DL2}$, and $T_{DL3}$ in Figure 16C are similar to those in Figure 15C. Therefore, compared to TPGFPlus-IND, THECB performs better in reducing the unpredictable electromagnetic interference on communication under varied network densities.

### 6.5. Performance of THECB with Varied Transmission Radius

The number of nodes is set as 160, and the proportion of nodes in different discharge levels is: $N_{DL1}$:$N_{DL1}$:$N_{DL1}$ = 4:3:3. The performance of the algorithm is evaluated under different transmission radius (set as 90 m, 100 m, 110 m, and 120 m, respectively).

**Figure 17:**
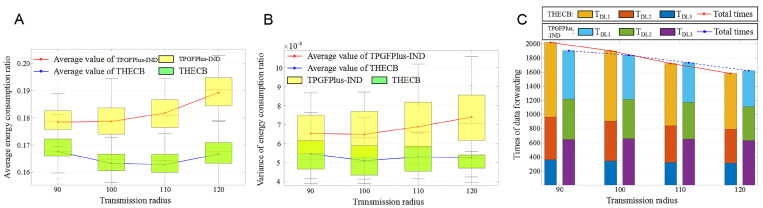
The performance of these algorithms with varied transmission radius. (**A**) Total energy consumption ratio. (**B**) Variance of energy consumption ratio. (**C**) The times of participating in data forwarding.

Figure 17A shows that $E_{rat}$ can be reduced by 6.15%, 8.38%, 10.38%, and 11.64% compared with TPGFPlus-IND under different transmission radius, which indicates that THECB performs better in saving energy consumption of communication.

$E_{var}$ in THECB and TPGFPlus-IND with the varied transmission radius are described in Figure 17B. It is noticed that compared to TPGFPlus-IND, our algorithm has up to 15.38%, 23.08%, 21.49%, and 29.73% lower variance, respectively. Thus, THECB performs better in the energy consumption balance of communication.

It is clear to see that in Figure 17C, as the radius increases, the sum of $T_{DL1}$, $T_{DL2}$ and $T_{DL3}$ becomes smaller. The main reason is that a longer radius leads to more neighbors, which are further to the current forwarder but nearer to the sink, to be chosen as the next-hop nodes. Furthermore, it would shorten the length of the paths to transmit data and reduce the data forwarding traffic simultaneously. In addition, same as Figure 15C and Figure 16C, THECB uses fewer nodes whose discharge numbers are relatively higher than those of other nodes to forward data and has less unpredictable electromagnetic interference on communication compared with TPGFPlus-IND.

### 6.6. Execution Demonstration of THECB and TPGFPlus-IND

**Figure 18:**
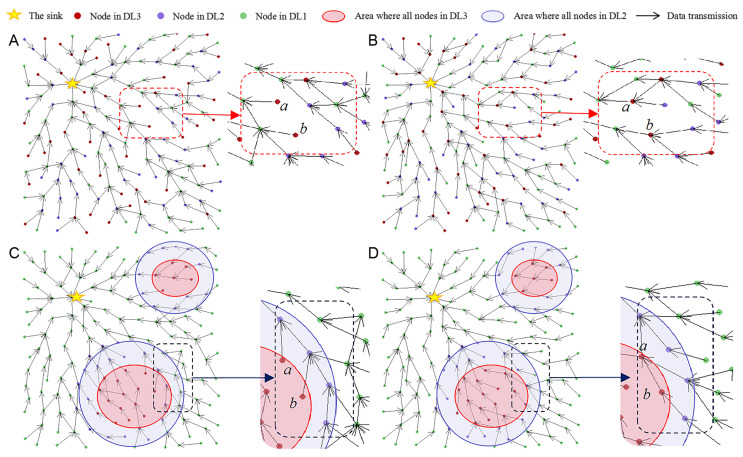
Compared to nodes of TPGFPlus-IND, those of THECB prefer to select the nodes in DL2 and DL1 to relay data, that is, nodes in DL3 have less data forwarding traffic, e.g., node *a* and *b*. (**A**) Node *a* and *b* of THECB are not selected as next hop nodes. (**B**) Node *a* and *b* of TPGFPlus-IND are selected as next hop nodes. (**C**) Node *a* and *b* of THECB are not selected as next hop nodes. (**D**) Node *a* and *b* of TPGFPlus-IND are selected as next hop nodes.

Figure 18 shows the execution result of THECB and TPGFPlus-IND. In Figure 18A and Figure 18B, the discharge numbers of different nodes are set randomly. In Figure 18C and Figure 18D, all nodes in a certain area are set to be in DL3 (the discharge number is relatively the highest). Nodes within a certain range around these nodes in DL3 are set to be in DL2 (the discharge number is relatively medium), and other nodes are set to be in DL1 (the discharge number is relatively the smallest). Compared to nodes of TPGFPlus-IND, those of THECB prefer to select the nodes in DL2 and DL1 to relay data; that is, nodes in DL3 have less data forwarding traffic.

Significantly, some nodes in DL3 are also selected to relay data in THECB. The main reasons are: (1) all the neighbors of the current forwarder are in DL3, so the next-hop node has to be selected from these neighbors, and (2) the current forwarder selects the next hop node based on not only the discharge number but also the distance from 2-hop neighbors to the sink and the ratio of communication energy consumption to residual energy. If the discharge number is high for the nodes on a path to be selected, but the distance or the ratio is small, the current forwarder may still choose a node in DL3 to be the next-hop node. Moreover, it is inappropriate to prohibit other nodes in DL2 and DL1 from selecting the nodes in DL3 to relay data to minimize the data forwarding traffic of the nodes in DL3, since bypassing some nodes to transfer data can make some paths extraordinarily long, which can increase the energy consumption of communication in the whole network.

## 7. Conclusions

In this paper, we propose THECB, a novel geographic routing algorithm based on 2-hop neighbors’ information, to improve the energy consumption balance and reduce the unpredictable electromagnetic interference on communication caused by the high voltage pulse current of communication paths SIL-IoTs. THECB contains three phases: neighbors’ information acquisition, geographic forwarding, and data dissemination. The greedy forwarding mechanism is expressed in the form of probability; that is, each neighbor node is given a weight between 0 and 1 according to the distance, and the weight represents the probability that the node is selected as the next-hop node. In the geographic forwarding method proposed, the nearby region around the current forwarder is divided into four parts according to the distance, and different weights are set to the nodes in different parts. Besides, different weights are set to the nodes in different discharge levels, thereby educating the data forwarding traffic of nodes whose discharge numbers are relatively higher than those of other nodes. The unpredictable electromagnetic interference in communication can be weakened. When exploring the path, the *routing-loop situation* and the *path-circle situation* will be solved if they occur. In addition, we calculate the energy consumed by one discharge of the SIL, which has not been investigated in the other works. Simulation results indicate that THECB achieves lower energy consumption, better energy consumption balance of communication, and less unpredictable electromagnetic interference of communication than TPGFPlus-IND (TPGFPlus ignores the requirement of the node-disjoint path) over a variety of discharge numbers, network densities, and transmission radius. Our scheme is designed for SIL-IoTs and the other IoT scenarios that have other energy consumption besides communication energy consumption and need to reduce the data forwarding traffic of some nodes.

## Figures and Tables

**Table 1:** Data Transmission Status in 2 Related Research Works of SIL-IoTs.

Year and Author	Data Transmission Model	Data Transmission between Nodes	Network Model	Optimized Energy Management
2013, H. Lam et al. [7]	Identify the species of pests through sensing the weight of pests in the SIL-IoTs nodes and transmit the pests data by time-division control, which contributes to establishing a real-time pests monitoring system	✓	Mesh network	×
2015, Qiu et al. [8]	The microcontroller in the node calculates the on-off time, and the data are transmitted to the node with the radar sensor by the wireless communication module	Partly	Star network	×

**Table 2:** Two-Hop Neighbors Information Based Algorithms.

Year	2012	2013	2016	2019	2020
Author	Singh et al. [9]	Congduc et al. [11]	Phonepadith et al. [10]	Hu et al. [13]	Zhang and Cai [12]
The main routing algorithm	TIE-GeR makes forwarding node selections via calculating the distance of the sink, link quality and residual energy of nodes	T-GPSR selects the most appropriate set of forwarding nodes for image transmission. based on the capture rate of image sensor nodes and the number of the current forwarder’s 1-hop and 2-hop potential forwarders	TN-CMAD calculates the average distance from 2-hop neighbors of the current forwarder to the sink and chooses 1-hop neighbor node that has the minimum value of this average distance as the next-hop node.	The forwarding node selection policy is according to the link quality determined by a new measure of minimum summation angle	EEPDBR evaluates node’s depth, residual energy, and forwarding number within its 2-hop neighbors to make routing decisions
Designed for WSNs	✓	✓	✓	✓	✓(Underwater Wireless Sensor Networks)
Belonging to geographic routing	✓	✓	✓	✓	✓(Depth Based Routing)
Data type	Not specify	Image data	Not specify	Not specify	Not specify
Energy consumption	Consider	Not consider	Not consider	Not consider	Consider
Interference of the communcation	Not consider	Not consider	Not consider	Not consider	Not consider
Optimization objectives	Achieve effective energy balancing throughout the network, while preventing routing voids by proactively avoiding “local maxim” nodes	Significantly reduce congestion and increase image quality at the robot sink when simultaneous images are sent from image sensor nodes	Reduce overhead packets and end-to-end delay and improve reachability	Reduce transmission overhead and end-to-end delay against communication voids in infrastructure-less environments	Optimize the transmission of sensory data with the help of depth information to improve packet delivery ratio (PDR) and energy efficiency
Network model	Not specify	Mobile sinks and fixed sensor, not specify the network density	Mobile Ad hoc networks, not specify the network density	Sparse mobile Ad hoc networks	Mobile Ad hoc networks, not specify the network density
Performance metrics of simulation	Packet delivery ratio and network lifetime	Packet loss rate, quality of received images at the robot sink and image transmission delay to the robot sink	Overhead (measured in packets), the packet delivery ratio, the average number of hops and the end-to-end delay	Delivery ratio and overhead ratio	Delivery time, packet delivery ratio and energy efficiency

**Table 3:** The comparison of TPGF, TPGFPlus and THECB.

Algorithm	Two-Phase Geographic Greedy Forwarding (TPGF) [15]	Two-Phase Geographic Greedy Forwarding Plus (TPGFPlus) [14]	Two-Hop Energy Consumption Balanced Routing (THECB)
Forwarding mechanisms	A forwarding node always chooses the next-hop node that is closest to the sink among all its neighbors, the next-hop node can be farther to the sink than itself	TPGFPlus chooses the next-hop node among its 1-hop and 2-hop neighbors according to three policies. If the chosen node is among 2-hop neighbors, an intermediate 1-hop direct neighbor needs to be selected	The nearby region around the current forwarder is divided into four parts according to the direction of transmitted packets. The nodes are graded into three levels according to the discharge number. The result of weighted summation of the distance from the 2-hop neighbors to the sink and the residual energy of that neighbor is also considered
The method of solving the *routing-loop situation*	*Steps Back and Mark* method: In the order from the node that finds this situation to the source node, nodes on the built path attempt to find a newly available path		The nodes explore the new path according to the sort of the distances from these nodes to the sink
The method of solving the *path-circle situation*	*Label Based Optimization* method: Each node is assigned a label. An acknowledgment is sent back to the source node after the path is found. Any node only relays the acknowledgment to its 1-hop neighbor based on the label		During exploring the routing path, a node *u* finding this situation sends a message packet to its 1-hop neighbor to inform it to update the routing table and transmit data packets to node *u* directly

**Table 4:** The important parameters.

Symbol	Meaning	Symbol	Meaning
$P_{L}$	The rated power of the lure lamp	ϕ	The sum of $\mu^{b_{1}}$ and $\mu^{b_{1}}$
$E_{L}$	The energy consumption of the lure lamp	*E*	The energy consumption factor
SATE1	The state when the SIL only turns on the lure lamp	α	The weight factor that determines the relative significance placed on $D_{sink}^{b_{2}}$ and *E*
SATE2	The sate when the SIL turns on both the lamp and the discharges simulation module	$e_{a}$	The energy consumed by communication of node a
$E_{SATE1}$ and $E_{SATE2}$	The energy consumption in SATE1 and SATE2 when the voltage changes from U1 to U2	$E_{res}$	The residual energy
T1 and T2	The working time in SATE1 and SATE2 when the voltage changes from U1 to U2	*A*	All the nodes of the found path
$E_{D}$	The energy consumption of one discharge	$A_{Loop}$	The nodes of the routing loop
*u*	The current forwarder	ρ	The mean number of neighbors per node
$D_{b}^{a}$	The distance from node *a* to node *b*	TR	The transmission radius
$\beta_{1}$, $\beta_{2}$, $\beta_{3}$ and $\beta_{4}$	The the weight factor of the four regions (mentioned in Section 4.3)	$E_{rat}$	Total energy consumption ratio
DL1, DL2 and DL3	The discharge number is relatively the smallest, medium and the highest	$E_{var}$	Variance of energy consumption ratio
$\mu_{1}$, $\mu_{2}$ and $\mu_{3}$	The weight of the node in DL1, DL2 and DL3	$N_{DL1}$, $N_{DL2}$ and $N_{DL3}$	The ratio of the number of nodes in DL1, DL2 and DL3
$\mu^{b_{1}}$ and $\mu^{b_{1}}$	The weight factor μ of node $b_{1}$ and $b_{2}$	$T_{DL1}$, $T_{DL2}$ and $T_{DL3}$	The data forwarding traffic of the nodes in DL1, DL2 and DL3

**Table 5:** The value of important parameters.

Parameter	Value	Parameter	Value
Network area	800 m ×800 m	$\mu_{1}$, $\mu_{2}$ and $\mu_{3}$	0.2, 0.3 and 0.5
the numeber of nodes	100 to 250	$\beta_{1}$, $\beta_{2}$, $\beta_{3}$ and $\beta_{4}$	0.05, 0.15, 0.3 and 0.5
TR	90 m to 120 m	$\varepsilon_{fs}$	10 pJ∖bit∖m${}^{2}$
the discharge number of nodes in DL1	300 to 1500	$E_{cir}$	50 nJ∖ bit
the discharge number of nodes in DL2	1500 to 2250	$\varepsilon_{amp}$	0.0013 pJ∖bit∖m${}^{4}$
the discharge number of nodes in DL3	2250 to 3000	$d_{0}$	87.7 m

**Table 6:** The proportion of different discharges levels in the 15 cases.

Case	1	2	3	4	5	6	7	8	9	10	11	12	13	14	15
$N_{DL3}$	1	1	1	1	1	3	3	3	3	5	5	5	7	7	9
$N_{DL2}$	1	3	5	7	9	1	3	5	7	1	3	5	1	3	1
$N_{DL1}$	8	6	4	2	0	6	4	2	0	4	2	0	2	0	0

## Data Availability

Not applicable.

## Abbreviations

The following abbreviations are used in this manuscript:

TPGFPlus	Two-Phase Geographic Greedy Forwarding plus
TPGFPlus-IND	TPGFPlus Ignores Node-Disjoint
IoTs	Internet of Things
SIL	Solar Insecticidal Lamp
SIL-IoTs	Solar Insecticidal Lamps Internet of Things
GSM	Global System for Mobile Communications
WSNs	Wireless Sensor Networks
PDR	Packet Delivery Ratio
SBM	Step Back and Mark
DEDST	Distance, Energy, and Discharge level Search Tree

## References

[1] Liu Y., Ma X., Shu L., Hancke G.P., Abu-Mahfouz A.M. (2021). From Industry 4.0 to Agriculture 4.0: Current Status, Enabling Technologies, and Research Challenges. IEEE Trans. Ind. Inform..

[2] Yang X., Shu L., Chen J., Ferrag M.A., Wu J., Nurellari E., Huang K. (2021). A Survey on Smart Agriculture: Development Modes, Technologies, and Security and Privacy Challenges. IEEE/CAA J. Autom. Sin..

[3] Li K., Shu L., Huang K., Sun Y., Yang F., Zhang Y., Huo Z., Wang Y., Wang X., Lu Q. (2019). Research and prospect of solar insecticidal lamps Internet of Things. Smart Agric..

[4] Han G., Dong Y., Guo H., Shu L., Wu D. (2015). Cross-layer optimized routing in wireless sensor networks with duty cycle and energy harvesting. Wirel. Commun. Mob. Comput..

[5] Huang K., Li K., Shu L., Yang X., Gordon T., Wang X. (2020). High Voltage Discharge Exhibits Severe Effect on ZigBee-Based Device in Solar Insecticidal Lamps Internet of Things. IEEE Wirel. Commun..

[6] Huang K., Li K., Shu L., Yang X. Demo Abstract: High Voltage Discharge Exhibits Severe Effect on ZigBee-based Device in Solar Insecticidal Lamps Internet of Things. Proceedings of the IEEE INFOCOM 2020—IEEE Conference on Computer Communications Workshops (INFOCOM WKSHPS).

[7] Lam B.H., Phan T.T., Vuong L.H., Huynh H.X., Pottier B. (2013). Designing a brown planthoppers surveillance network based on wireless sensor network approach. arXiv.

[8] Qiu Z., Qiu P., Zhu Q., Wang R. (2015). Insect Trapping Method Based on Progressive Star Network. Int. J. Online Biomed. Eng..

[9] Singh I.B., Ho Q.D., Le-Ngoc T. TIEGeR: An Energy-Efficient Multi-Parameter Geographic Routing Algorithm. Proceedings of the 2012 IEEE Vehicular Technology Conference (VTC Fall).

[10] Phoummavong P., Utsu K., Chow C.O., Ishii H. (2016). Location-aided route discovery mechanism based on two-hop neighbor information for ad hoc network. J. Supercomput..

[11] Pham C., Diop E.H.S.M., Thiare O. (2013). Selecting source image sensor nodes based on 2-hop information to improve image transmissions to mobile robot sinks in search & rescue operations. arXiv.

[12] Zhang M., Cai W. (2020). Energy-Efficient Depth Based Probabilistic Routing Within 2-Hop Neighborhood for Underwater Sensor Networks. IEEE Sens. Lett..

[13] Hu C.L., Sosorburam C. (2019). Enhanced Geographic Routing with Two-Hop Neighborhood Information in Sparse MANETs. Wirel. Pers. Commun..

[14] Han G., Dong Y., Shu L., Guo H., Niu J. Geographic Multipath Routing in Duty-Cycled Wireless Sensor Networks with Energy Harvesting. Proceedings of the 2013 IEEE International Conference on Green Computing and Communications and IEEE Internet of Things and IEEE Cyber, Physical and Social Computing.

[15] Shu L., Zhang Y., Yang L.T., Wang Y., Hauswirth M., Xiong N. (2010). TPGF: Geographic routing in wireless multimedia sensor networks. Telecommun. Syst..

[16] Yang F., Shu L., Huang K., Li K., Han G., Liu Y. (2020). A Partition-Based Node Deployment Strategy in Solar Insecticidal Lamps Internet of Things. IEEE Internet Things J..

[17] Yang X., Shu L., Huang K., Li K., Yao H. Poster Abstract: Insecticidal Performance Simulation of Solar Insecticidal Lamps Internet of Things Using the Number of Falling Edge Trigger. Proceedings of the IEEE INFOCOM 2021—IEEE Conference on Computer Communications Workshops (INFOCOM WKSHPS).

[18] Shu L., Hauswirth M., Chao H.C., Chen M., Zhang Y. (2011). NetTopo: A framework of simulation and visualization for wireless sensor networks. Ad Hoc Netw..

[19] Huang H., Yin H., Min G., Zhang J., Wu Y., Zhang X. (2018). Energy-Aware Dual-Path Geographic Routing to Bypass Routing Holes in Wireless Sensor Networks. IEEE Trans. Mob. Comput..

[20] Zhao X.Q., Cui Y.P., Gao C.Y., Guo Z., Gao Q. (2020). Energy-Efficient Coverage Enhancement Strategy for 3-D Wireless Sensor Networks Based on a Vampire Bat Optimizer. IEEE Internet Things J..

[21] Huang S.C., Lo Y.L., Lu C.N. (2013). Non-Technical Loss Detection Using State Estimation and Analysis of Variance. IEEE Trans. Power Syst..

